# Changes in the vaginal microbiota following antibiotic treatment for *Mycoplasma genitalium*, *Chlamydia trachomatis* and bacterial vaginosis

**DOI:** 10.1371/journal.pone.0236036

**Published:** 2020-07-28

**Authors:** Peter Ahrens, Lee O’Brien Andersen, Berit Lilje, Thor Bech Johannesen, Ebba Gomez Dahl, Sharmin Baig, Jørgen Skov Jensen, Lars Falk

**Affiliations:** 1 Department of Bacteriology, Parasitology and Mycology, Statens Serum Institut, Copenhagen, Denmark; 2 Department of Dermatovenereology, Linköping University Hospital, Linköping, Sweden; 3 Department of Biomedical and Clinical Sciences, Linköping University, Linköping, Sweden; University of California, San Francisco, Universit of California, Berkeley and the Childrens Hospital Oakland Research Institute, UNITED STATES

## Abstract

The human vagina harbor a rich microbiota. The optimal state is dominated by lactobacilli that help to maintain health and prevent various diseases. However, the microbiota may rapidly change to a polymicrobial state that has been linked to a number of diseases. In the present study, the temporal changes of the vaginal microbiota in patients treated for sexually transmitted diseases or bacterial vaginosis (BV) and in untreated controls were studied for 26 days. The patients included 52 women treated with azithromycin, tetracyclines or moxifloxacin for present or suspected infection with *Chlamydia trachomatis* or *Mycoplasma genitalium*. Women with concurrent BV were also treated with metronidazole. The controls were 10 healthy women of matching age. The microbiota was analyzed by 16S rRNA gene deep sequencing, specific qPCRs and microscopy. There was generally good correlation between Nugent score and community state type (CST) and qPCR confirmed the sequencing results. By sequencing, more than 600 different taxa were found, but only 33 constituted more than 1 ‰ of the sequences. In both patients and controls the microbiota could be divided into three different community state types, CST-I, CST-III and CST-IV. Without metronidazole, the microbiota remained relatively stable regarding CST although changes were seen during menstrual periods. Administration of metronidazole changed the microbiota from CST-IV to CST-III in approximately 50% of the treated patients. In contrast, the CST was generally unaffected by azithromycin or tetracyclines. In 30% of the BV patients, *Gardnerella vaginalis* was not eradicated by metronidazole. The majority of women colonized with *Ureaplasma parvum* remained positive after azithromycin while *U*. *urealyticum* was eradicated.

## Introduction

The vaginal microbiota plays an important role in health and disease in reproductive age women. It is widely accepted that lactic acid producing *Lactobacillus* species is a major factor for maintenance of an environment unfavorable for various pathogenic bacteria, and thereby of importance for vaginal health. Depletion of lactobacilli may predispose to adverse conditions as preterm birth, acquisition of sexually transmitted infections and pelvic inflammatory disease. Further, depletion of Lactobacilli may lead to bacterial vaginosis (BV), a condition that is characterized by a more diverse microbiota consisting of *Gardnerella vaginalis*, *Atopobium vaginae*, *Megasphaera* and number of other anaerobe or facultative anaerobe bacterial species [1].

To characterize the vaginal microbiota Ravel *et al* introduced the term community state types (CSTs) where CST-I; CST-II, CST-III and CST-V were dominated by lactobacilli whereas CST-IV showed a higher diversity and was dominated by a mix of mainly anaerobic bacteria [2] and related to BV.

The prevalence of BV varies in different populations. American studies have found the highest prevalence in women of African descent and lower in women of European and Asian origin [3]. Whether these observed differences are caused mainly by genetic factors or also related to socio-economic factors, is not fully understood. Recent studies have found BV related to birth control methods and other factors as menstrual hygiene practices [4], obesity [5] and level of education [6]. A CST-IV vaginal microbiota has also been linked to preterm birth [7] and decreased embryo implantation success in assisted reproduction [8,9].

Earlier longitudinal studies have shown the vaginal microbiota to be highly variable, fluctuating with menstrual period, pregnancy and sexual activity, but also remarkably stable in a number of women [2,10,11]

In Western countries, *Chlamydia trachomatis* and *Mycoplasma genitalium* are the most common sexually transmitted infections (STIs); BV has been linked to gonorrhea and chlamydia in earlier studies [12] and has also been associated with *M*. *genitalium* acquisition [13]. More recent studies have associated Lactobacilli with protection against chlamydia [14], and immune mediators appears to be of importance [15].

Few longitudinal studies of the vaginal microbiota during treatment with antibiotics have been conducted.

Here we studied the dynamics of the vaginal microbiota for 26 days in patients treated for *C*. *trachomatis* or *M*. *genitalium* infection with and without BV. In addition, matched healthy untreated controls were included. The purpose was to estimate the changes in the vaginal microbiota caused by antibiotics for treatment of STIs and BV.

## Materials and methods

### Patients

Patients in this study were participants in a prospective longitudinal cohort study comprising an observational study and a randomized treatment trial aimed at determining the time to eradication of *M*. *genitalium* [16]. The present study includes additional women not reported in the primary study. A total of 52 patients were selected from women attending two sexually transmitted disease clinics between April 2010 and July 2015. The patients were attending two STD-clinics (Norrköping or Linköping, Sweden) and asked for participation if they were contacts *M*. *genitalium* infected patients or if they had symptoms requiring antibiotic treatment. Group 1 comprised patients with a high risk of *M*. *genitalium* infection, i.e. with a current partner having an *M*. *genitalium* infection or patients not treated initially, but with a positive *M*. *genitalium* test. These patients (n = 30) were treated with extended azithromycin (500 mg day 1 followed by 250 mg on days 2–5) which is the recommended treatment for *M*. *genitalium* in Sweden. It was deemed unethical to randomize these patients. If there was evidence of macrolide resistance, moxifloxacin (400 mg x 1 for 7 days) was prescribed (n = 2). Group 2 comprised symptomatic patients with no known exposure to *M*. *genitalium*. These patients were randomized to receive tetracyclines, the standard of care in Sweden (doxycycline 200 mg on day 1 and 100 mg once daily on days 2–9; a total of 1 g (n = 5) or alternatively, during summer, to decrease the risk of photosensitization lymecycline 300 mg twice daily for 10 days (n = 1)) or to a 1 g single dose of azithromycin (used for syndromic treatment of urethritis/cervicitis), on the day of the first visit (n = 14). A substantial proportion of these patients had a chlamydial infection or idiopathic urethritis and/or cervicitis. Furthermore, 10 healthy controls of matching age (recruited among university students in the same area, Linköping, Sweden) were included.

In the clinic, patients underwent a genital examination, including evaluation of bacterial vaginosis (BV) by Amsel criteria [17] with microscopy of vaginal wet smears. Methylene blue stained smears from the endocervix and urethra were examined by microscopy for cervicitis and urethritis. An additional endocervical/vaginal swab was collected in GeneLock DNA preserving transport medium (Sierra Diagnostics, Sonora CA, USA). Women fulfilling Amsel’s criteria for BV, were prescribed treatment with oral metronidazole (400 mg bid for 7 days (n = 16) or a 2g dose at the day of the gynecological examination (day 0) and at day 2(n = 6)). Two women did not receive metronidazole despite clinical BV; the reason was not noted. Six women only collected self-obtained vaginal swab samples at the day of inclusion, and no genital examination was performed. The 10 untreated women participating as controls did not undergo genital examination either.

All participants were provided with 2x12 Copan flocked swabs (Copan, Brescia, Italy), 12 tubes with GeneLock transport medium, and the patients also 12 glass slides with transport covers and given instructions on how to collect vaginal smears properly by inserting two sterile flocked swabs 3–4 cm into the vagina, using the breaking score as a guide. At home, vaginal swab samples were collected at day 1, 3, 5, 8, 10, 12, 15, 17, 19, 22, 24 and 26. One swab was used for DNA preparation, the other rolled on a glass slide for microscopy. Samples were sent to Statens Serum Institut (SSI) in Copenhagen, Denmark at weekly intervals. The glass slides were heat fixed and Gram-stained at SSI and BV was scored according to Nugent [18].

From the 10 controls, all 12 samplings were used for sequencing, from patients, only the samples from days 0, 1, 3, 5, 8, 12, 19 and 26 were sequenced due to limited sequencing capacity.

The patient’s age, symptoms, treatment, anticonception, number of sexual partners (lifetime and during the last 12 months) were recorded (shown in Table 1).

**Table 1:** Characteristics of the included patients and controls.

Characteristic	Controls	Patients	p
	(n = 10)	(n = 52)	
*Age in years*	23 (22–30)	23(16–53)	ns
No. sex partners, past 12 months	1(0–8)	3 (1–20)	0.041
No. life-time sex partners	12.5 (2–30)	10.5 (1–150)	ns
*Sexual preference*			
WSM	10	50	ns
WSW	0	2	
*Symptoms and signs*		(n = 46)	
Bacterial vaginosis	-	24 (52%)	
Cervicitis	–	27 (59%)	
Urethritis	–	12 (26%)	
Urethritis Intermediate	–	10 (22%)	
*Treatment*		(n = 52)	
Azithromycin	-	44 (85%)	
Tetracycline	-	6 (12%)	
Moxifloxacin	-	2 (4%)	
Metronidazole	-	22 (42%)	
*Infection*			
*C*. *trachomatis*	-	10 (19%)	
*M*. *genitalium*	-	32 (62%)	
*M*. *genitalium and C*. *trachomatis*	-	2 (4%)	
*M*. *genitalium* and *C*. *trachomatis* negative *Contraception*	10	8 (15%)	
Hormone, combined	3 (30%)	18 (35%)	ns
Hormone, gestagen only	3 (30%)	13 (25%)	ns
Other (copper intra uterine device)	1 (10%)	1 (2%)	ns
None, condom	3 (30%)	19 (37%)	ns
NA	0	1 (2%)	ns

### Extraction of DNA

From 100 μL of transport medium, vaginal swab DNA was extracted with the FastDNA™ spin kit for soil as recommended by the supplier (MP biomedicals, Santa Ana, Ca, USA) and subsequently eluted in 100 μL DNAse free water.

### qPCR

The total bacterial load was determined by qPCR using the same primers as used for sequencing of 16S rRNA genes (341F and 806R) as previously described [19]. Further, the load of selected bacterial species was determined by specific qPCR tests as previously described for *M*. *genitalium* [20] *C*. *trachomatis* [21] *U*. *urealyticum* and *U*. *parvum* [22], *A*. *vaginae* and *G*. *vaginalis* [23].

### Sequencing

The V3-V4 regions of the 16S rRNA gene, were PCR amplified and sequenced on the Illumina MiSeq platform using primers and the v2 reagent kit as earlier described [24,25]. All PCR amplifications included dilutions of *Legionella pneumophila* as positive control and, as negative controls, pure water samples (blanks) were included. All PCR preparations were performed in laminar flow hoods in dedicated laboratories to minimize environmental contamination.

### Data analysis

Sequences were mapped using BION, a k-mer-based software as earlier described [26]. All sequence runs included positive and negative controls) that went through DNA purification, PCR amplification and sequencing along with the clinical samples. Sequences from 10 of these blanks that contained a sufficient number of sequences were used for contaminant analysis. Contaminating bacterial sequences is a common problem in microbiota analysis, especially in low biomass samples [27]. To address this, species that were significantly negatively correlated to bacterial load and species that were dominant in blanks were considered contaminants and removed. A list of these contaminant species is shown in supportive S1 Table.

The cut off level for sequences for inclusion in the analysis was determined subjectively by balancing the number of samples versus sequencing depth. To enable direct comparison of samples with variation in number of sequences, these were rarefied to 5431 sequences per sample (the lowest sequencing depth of any included sample). The sequence data has been deposited in Genbank, Sequence Read Archive under SRA accession: PRJNA638549.

### Statistics

Fisher’s exact test, Mann-Whitney test, Pearson’s Correlation and Wilcoxon signed-rank test were done using the calculator at www.soscistatistics.com.

Pearson correlation (calculated in Excel) using Benjamini-Hochberg adjustment for false discovery rate [28] was used for analysis of possible contaminants.

PCoA plots, Shannon diversity calculation and plots, heatmaps and barplots were generated in R version 3.5.0 [29]. For the heat map Bray-Curtis dissimilarity and Ward clustering was used.

A significance level of 5% and two-sided results was used in all tests.

### Ethics statement

All participants provided verbal consent after having received written and oral information regarding the study. The verbal consent was noted in their medical record by the researcher. The regional research ethics committee of Linköping, Sweden, approved the study (M 134–09, T126-09) and (Dnr 2016/87-31) including the verbal consent procedure (patients treated with antibiotics (2009 M134-09). All controls provided written informed consent (2016/87-31). According to the Biobanks in Medicine Care Act (2002:297) a biobank was registered in the Department of Dermatology and Venereology, Region Östergötland. The study was described in Clinical Trials Registration # NCT01661985.

## Results

### Patients and samples

A total of 52 patients and 10 controls were included in the study. Patient demographics are summarized in Table 1.

For treatment of diagnosed or presumed *M*. *genitalium* or *C*. *trachomatis* infection, all patients were prescribed either azithromycin (n = 44; 14 received 1g single dose, 30 extended 1.5g dose), tetracyclines (n = 6; 5 doxycycline, 1 lymecycline), or moxifloxacin (n = 2). For treatment of diagnosed BV, 22 patients were prescribed metronidazole.

There was no difference in the median age or number of lifetime sexual partners between patients and controls. Patients had, however, a significantly higher number of sex partners in the previous 12 months.

We observed no significant differences in contraceptive methods in patients or controls.

A number of samples were lost in the course of analysis: eight samples were missing or without biological material at arrival in the analyzing laboratory. Following quantification of the bacterial load by broad-range 16S qPCR, a cut off was set to 9.0E5 copies per mL of the original sample. Samples with lower DNA load were considered low biomass samples and discarded (n = 32).

**Fig 1:**
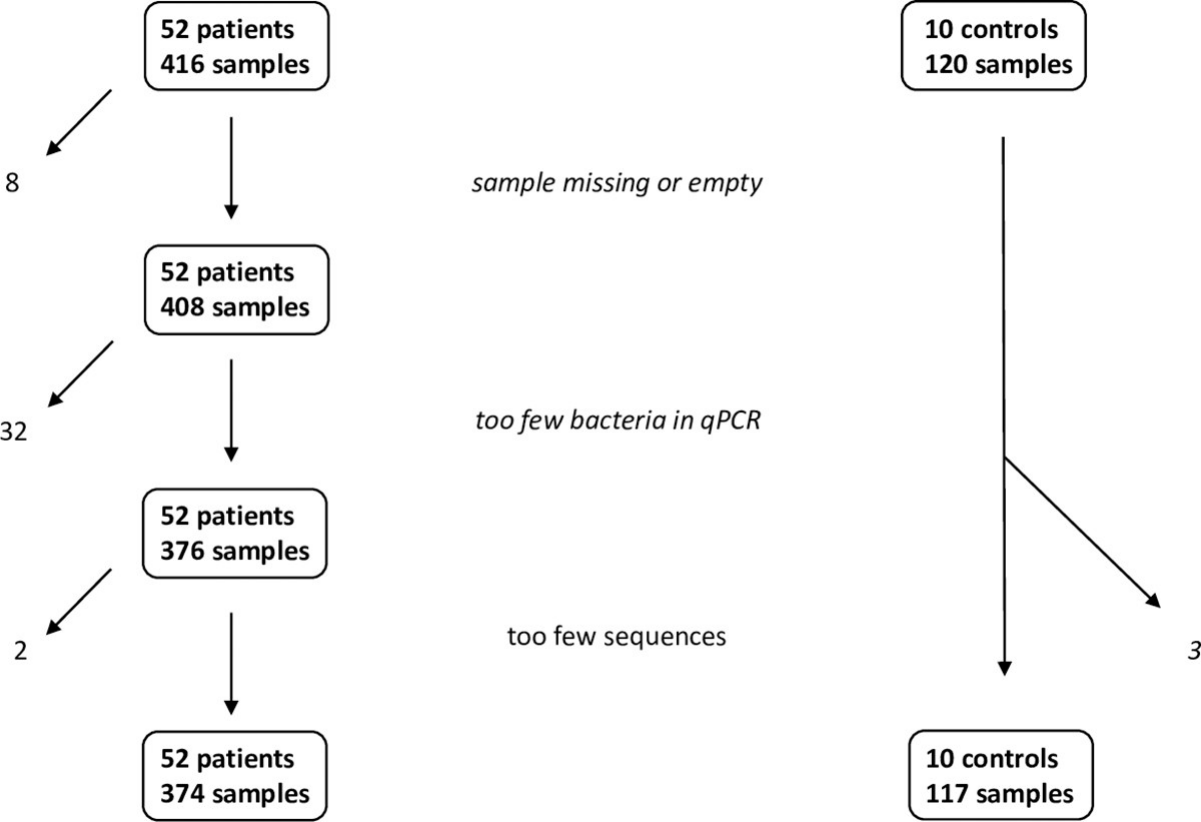
Flowchart of samples from 52 patients and 10 controls.

A minimum sequencing depth of 5000 sequences per sample was chosen for samples included in the analysis. This requirement was not met by two patient samples and three samples from controls. These filtering steps left 374 samples from 52 patients and 117 samples from 10 controls with a median load of 16S rRNA genes of 3.3E+08 (range 9.8E+5–6.7E+10) for analysis (Fig 1).

The 32 discarded samples were from 21 different patients. These were from the following samplings: Day 0: 5; day 1: 2; day 3: 3; day 5: 2; day 8: 7; day 12: 5; day 19: 4 and day 26: 4. The three discarded samples from controls were from day 3, 5 and 15 of three different controls.

### Sequences

A total of 29,249,395 sequences was obtained from the included samples. The samples had a median of 58,263 sequences (range 5,431–124,303), and all samples were rarefied to the minimum number of sequences (5431).

After filtering, the total number of species/taxa was 615 in the 491 patient and control samples. In patients, the median number of species was 23 (range 9–44) at day zero before treatment and 17 (range 3–77) for the days after treatment. In the control samples the median number of species was 19 (range 6–52).

The microbiota was diverse: 370 (58%) of the bacterial taxa were only present in three or fewer of the 491 samples and only 33 constituted more than 1 ‰ of all sequences. Furthermore, 340 of the 615 species (55%) were present with fewer than 10 copies in the complete rarefied dataset.

A number of sequences could not be classified to species, these were analyzed at a higher taxonomic level. In particular, *U*. *urealyticum* and *U*. *parvum* sequences were identical and shown as *Ureaplasma* spp, *Veillonellaceae* unclass included *Megasphaera* type 1, and all members of the family *Enterobacteriaceae* were combined into one taxon.

The distribution of species in patients and controls is shown in S2 Table.

### Abundant species before treatment with antibiotics

**Fig 2:**
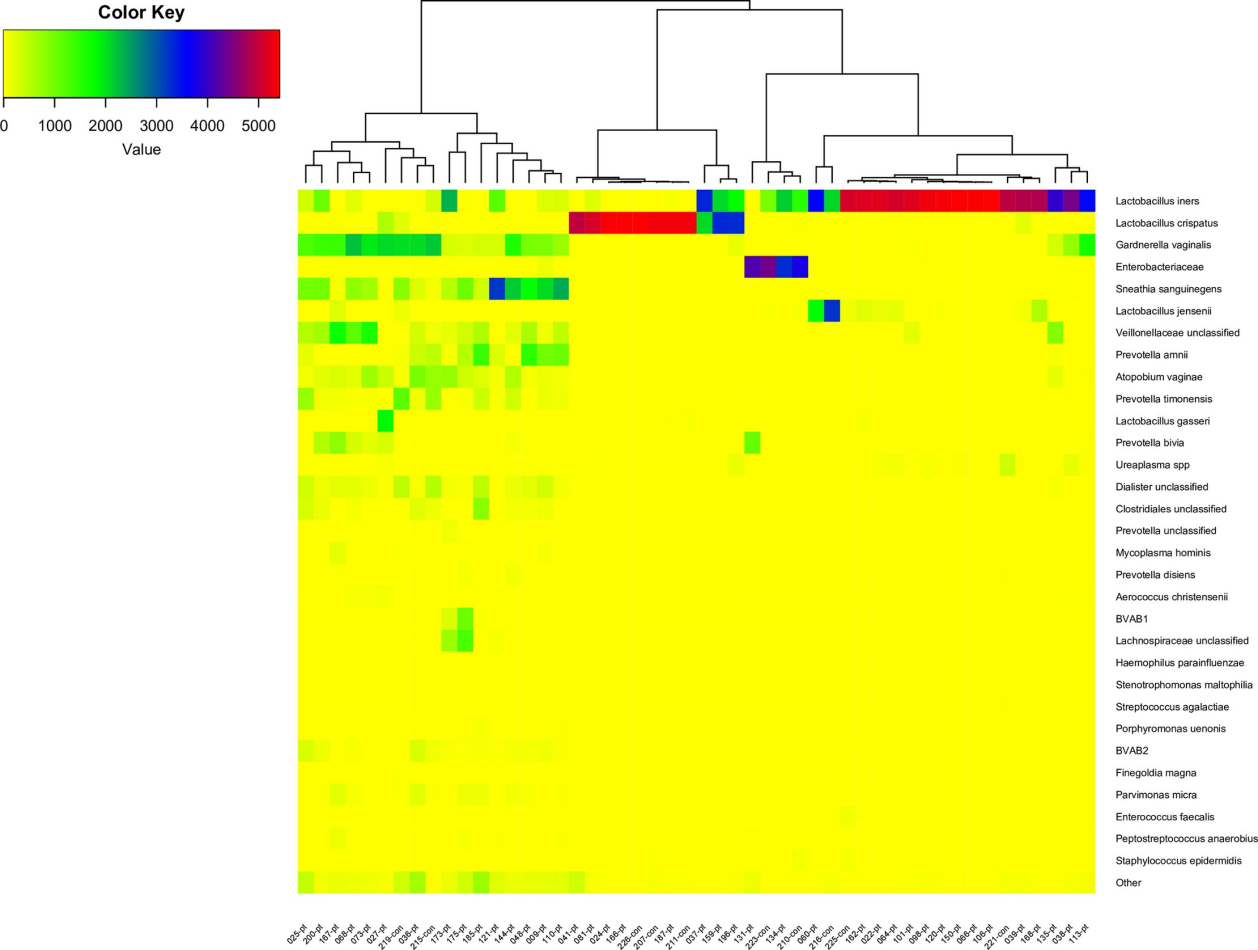
Heatmap of 31 dominant OTUs and the rest combined as “other” of sample 0 from the patients and sample 1 from the controls. The dendrogram show a hierarchical clustering of samples based on Bray-Curtis dissimilarity. The patient number and group (con = control, pt = patient) is shown on the x-axis.

The distribution of 31 of the 33 taxa each constituting more than 1‰ of the sequences (and the rest combined as “other”) for sample 0 from the patients and sample 1 from the controls is shown in the heat map in Fig 2.Two of the 33 dominant species, *Haemophilus haemolyticus* and *Achromobacter ruhlandii*, were not present in the first samples.

These 31 dominant taxa constituted 98.1% of all sequences in these first samples. The samples formed three main clusters corresponding to the common CSTs: CST-I, CST-III and CST-IV (Fig 2) [2]. One cluster of 11 samples dominated by *L*. *crispatus*, another of 22 dominated by *L*. *iners*, this cluster also included samples dominated by *Enterobacteriaceae*. Finally, a third cluster of 17 samples containing a mix of different species including *G*. *vaginalis*, *A*. *vaginae*, *Prevotella* and other BV associated bacteria. The median Shannon diversity index for the three clusters was 0.15, 0.34 and 1.93 respectively (p-values CST-I vs CST-III: NS, CST-I vs CST-IV: 0.00008, CST-III vs CST-IV: 0.00001, Mann-Whitney).

### Dominant species per patient

Only 10 taxa constituted more than 1% of the sequences: *L*. *iners* (41.0%*)*, *L*. *crispatus* (26.0%), *G*. *vaginalis* (6.5%), *Enterobacteriaceae* (3.5%), *Sneathia sanguinegens* (3.1%), *L*. *jensenii* (2.5%), *Veillonellaceae* unclass (1.9%), *Prevotella amnii* (1.7%), *A*. *vaginae* (1.7%) and *P*. *timonensis* (1.3%). In total, these ten taxa constituted 91.5% of all sequences.

**Fig 3:**
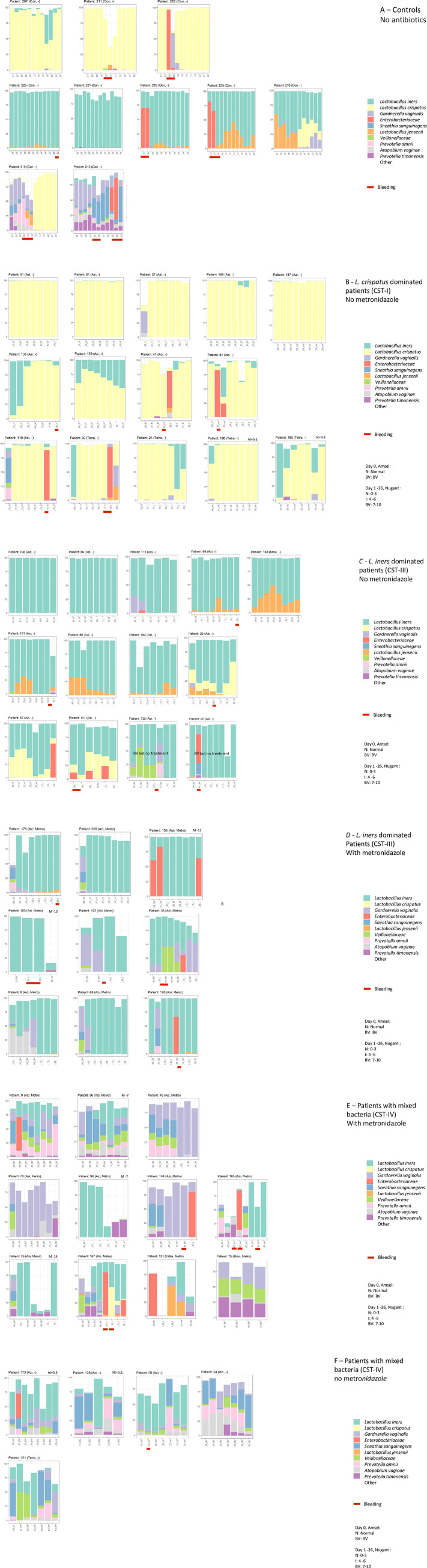
Bar plot of the ten most common bacterial species shown per patient. For each day the percentage for each of the 10 most prevalent species is shown. Other species appear combined as white in top of each bar. Above each bar plot is given the patient code and, in parentheses, the antibiotic used (Azi,): azithromycin, (Tetra,): tetracycline, (Moxi,): moxifloxacin. (, Metro): metronidazole. A few patients received metronidazole before day 0, this is indicated as M-11, M-7 etc. Patients without gynecological examination are marked “no G.E.”. Under each plot is given the day and bacterial vaginosis score (BV, intermediate or normal). Further days with bleeding are shown in red. A: Controls. Top panel CST-I, Middle panel: CST-III, bottom panel CST-IV. B: Patients with CST-I. C: Patients with CST-III not treated with metronidazole. D: Patients with CST-III, treated with metronidazole. E: Patients with CST-IV, treated with metronidazole. F: Patients with CST-IV, not treated with metronidazole.

To study the temporal changes of the microbiota, the presence of these 10 most prevalent species in vaginal swabs is shown per patient in Fig 3, where the patients are sorted according to the most dominant CST.

The controls and patients could be divided into three different profiles. 1) Those dominated (i. e. > 50% of sequences) by *L*. *crispatus* (corresponding to CST-I), 2) those dominated by *L*. *iners* (CST-III) and 3) those dominated by other bacteria (CST-IV) [2]. By this definition, a microbiota dominated by e.g. *Enterobacteriaceae* was classified as CST-IV.

In the controls, the microbiota was found to be relatively stable over time as shown in Fig 3A. Two of the three CST-I controls showed a stable, *L*. *crispatus* dominated microbiota that briefly changed during a menstrual period but rapidly returned to CST-I after the bleeding. In the five CST-III controls, two of these also showed a change in the microbiota related to bleeding, but otherwise showed a stable *L*. *iners* microbiota, but also supplemented with *L*. *jensenii*. Finally two controls had a microbiota initially dominated by mixed bacteria (CST-IV). In one of these controls (#219) the mixed microbiota completely shifted to one dominated by *L*. *crispatus* after a menstrual period. Interestingly, *L*. *crispatus* was present at low abundance in the CST IV-type microbiota before the menstrual bleeding, but absent from two samples during the bleeding.

In the patients (Fig 3B–3F), 14 (27%) were dominated by *L*. *crispatus* (Fig 3B). None of these patients were diagnosed with BV and most of the samples (71%) had a normal Nugent score. In three patients, the majority of samples had an intermediate Nugent score, and five other patients had one or more samplings showing an intermediate Nugent score. A number of patients had *L*. *iners* dominated (#103, #39, #190) or diverse microbiota (#27, #110) in their initial samples, but went on to *L*. *crispatus* dominated microbiota after treatment for their STI without metronidazole. In two other patients (#159, #24) there was an increase of *L*. *iners* in the late samples. Two patients (#190 and #196) had no gynecological examination and no initial evaluation; one patient (#187) had no follow-up evaluation. Only three patients (#61, #166 and #110) showed a normal Nugent score in all samples.

For 22 patients (42%), the microbiota was dominated by *L*. *iners*, and 11 showed no sign of BV at day 0 (Fig 3C), but all had one or more samplings with an intermediate Nugent score although most of the samples (58%) were scored as normal. In many of these patients, *L*. *jensenii* was also prominent. In three patients (#25, #37, #141) *L*. *crispatus* was present in all samples, but without a clear change in CST during the observation period. Two patients were diagnosed with BV, but not treated with metronidazole (#22 and #135). Despite no treatment, both of these patients changed to an *L*. *iners* dominated microbiota before the end of the study period (Fig 3C). In another 9 patients, the vaginal microbiota was also dominated by *L*. *iners*, but these patients were initially diagnosed with BV and treated for the condition with metronidazole. In all of these patients, *L*. *iners* became the dominant species shortly after treatment (Fig 3D). All of these patients had one or more BV positive samples and the majority with an intermediate Nugent score and only one patients (#134) had two samples that were Nugent 0–3. One patient (#64) was earlier (day -25) diagnosed with BV but not treated with metronidazole before day 0.

Finally, 16 patients (31%) were dominated by a microbiota of mixed bacteria, CST IV. Eleven of these 16 patients (Fig 3E) were treated with metronidazole but in 6/11 there was no change of the Nugent score during the study period. In another five patients, there was some effect of the antibiotic treatment, but no sample from any of the patients reached a Nugent score of 0–3. Five patients with mixed bacteria were not treated with metronidazole (Fig 3F): Three patients had no gynecological examination, another two (#50, #121) were Amsel BV negative in the clinic at day 0, but BV positive by Nugent in the home-collected samples (Fig 3F).

Across the CST groups, short periods dominated by *Enterobacteriaceae* was observed in both patients and controls. In a most of the patients, these temporary changes in the microbiota were seen in conjunction with periods of bleeding (Fig 3). Also the Shannon diversity index was higher during bleeding (0.94 vs 0.44; p = 0.00008 Mann-Whitney).

### Antibiotics, load and diversity

A reduction in the bacterial load following treatment with metronidazole and other antibiotics, could be expected. However, there was no significant reduction in the median total bacterial load, as determined by broad range 16S rRNA gene qPCR in patient samples from day zero and any of the following sampling days (S1 Fig, p = 0.66, 0.7, 0.94, 0.12, 0.26, 0.07 and 0.4, respectively, Wilcoxon).

In contrast to the total bacterial load, the diversity before and after treatment differed.

**Fig 4:**
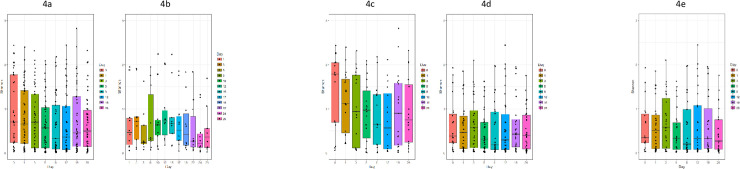
Boxplot of Shannon diversity index given per sampling. a: All patients. b: All controls. c: Patients treated with metronidazole. d Patients without metronidazole treatment. e: Patients initially positive for *C*. *trachomatis* or *M*. *genitalium* and not treated with metronidazole.

When comparing diversity in patients according to sampling time, there was a trend towards a declining Shannon index from day 0 to day 12), followed by an increase at day 19 and 26, shown in Fig 4A.

However, only at day 8 and 12 the index was statistically significantly lower than at day 0 (p< 0.002 and p< 0.03, respectively, Wilcoxon).

When comparing controls and patients treated with azithromycin, tetracyclines and moxifloxacin respectively, there was no significant difference in Shannon index between these groups (not shown).

Patients diagnosed with BV had a more diverse microbiota than patients without this diagnosis. At days 0–8 and 26 the diversity was higher in metronidazole treated BV patients than in patients without metronidazole treatment, including patients without BV. However, only at days 0, 1, 5 and 26 the difference was statistically significant (p = 0.006, 0.007, 0.02 and 0.005, respectively; Mann-Whitney (Fig 4C and 4D). For the five patients diagnosed with BV but without metronidazole treatment, the Shannon index was between 0.72 and 2.45, i.e. similar to day zero for the metronidazole treated patients, but without a reduction over time as seen in the treated patients.

For patients not treated with metronidazole, there was no statistically significant different decrease in diversity in relation to the antimicrobial treatment (Fig 4D). This was true regardless of the antimicrobial class although too few patients received tetracyclines or moxifloxacin to allow meaningful conclusions.

Nineteen (37%) of the patients were treated for a presumed STI based on symptoms and/or signs of urethritis or cervicitis. The remaining patients (63%) were treated as contacts to *M*. *genitalium* or *C*. *trachomatis* positive partners or due to a positive diagnostic test for one of the two STIs. It could be speculated, that the inflammation caused by STIs could promote a shift in the microbial diversity. However, for patients positive for *C*. *trachomatis* or *M*. *genitalium*, but without metronidazole treatment, there was no statistically significant difference in diversity before or after treatment for *M*. *genitalium* and *C*. *trachomatis* (Fig 4E).

### Bacterial vaginosis and microbiota

In the clinic, 24 of the 52 patients were diagnosed with BV by Amsel criteria. Another two patients (#50 and #121) were negative by Amsel at day 0 but were found BV positive by Nugent all the following days. These two patients had a mixed CST-IV microbiota, also at day 0.

**Fig 5:**
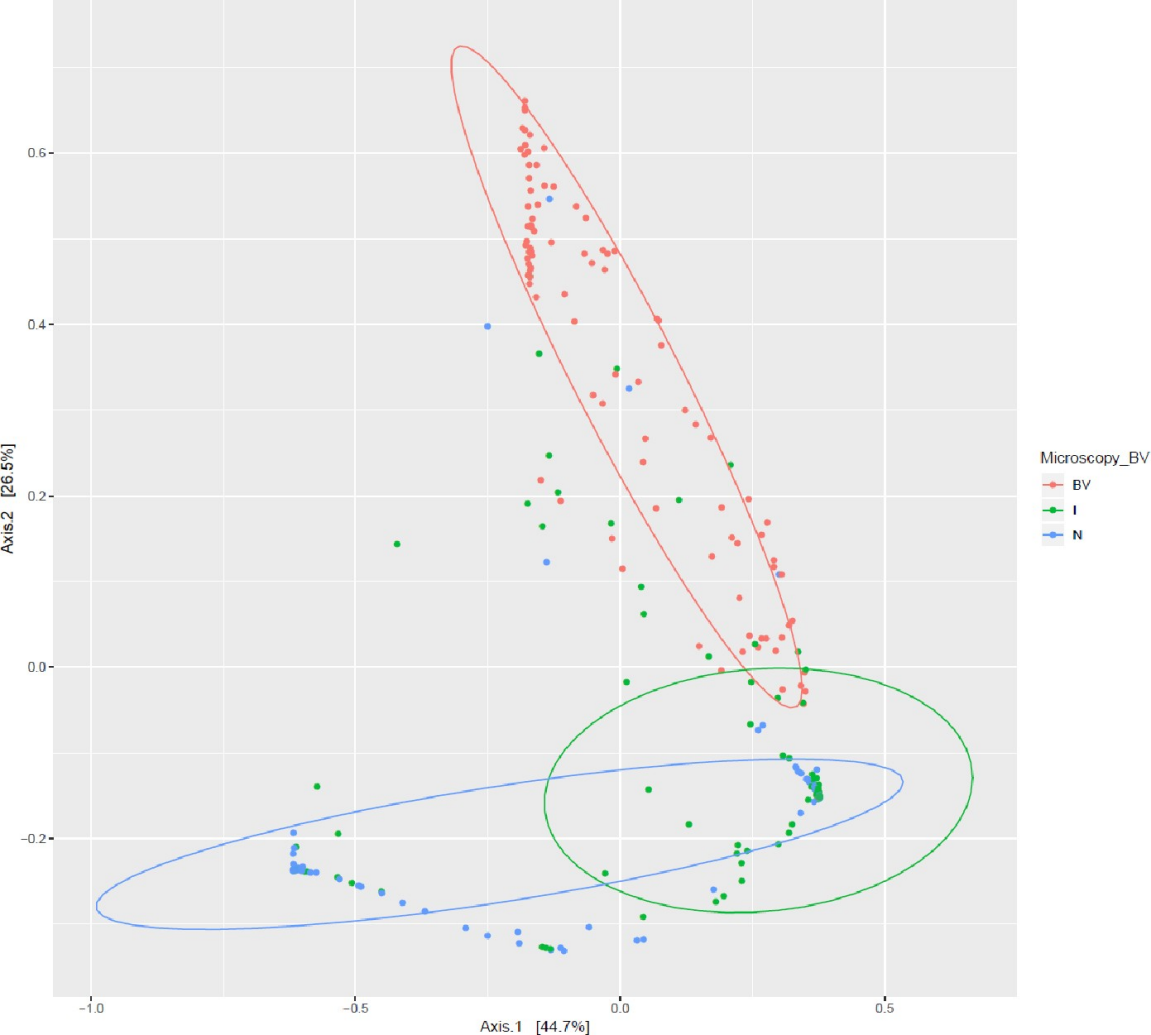
PCoA plot the 277 patient samples with a Nugent score. Clustering based on Bray Curtis dissimilarity calculated from taxonomic composition and colored according to Nugent score: Green = normal (Nugent 0–3), blue = intermediate (Nugent 4–6) and red = BV (Nugent score 7–10).

The relation between the microbiota and Nugent score is displayed in the PCoA plot in Fig 5, showing a good correlation between the two different methods.

Of the 22 patients treated with metronidazole, all were qPCR positive for *G*. *vaginalis*. However, two of the 22 patients were treated one and two weeks respectively before day 0 and their samples had low organism load, so the effect of metronidazole could not be evaluated. In 11 (58%) of the evaluable 19 patients, *G*. *vaginalis* was eradicated or significantly reduced (more than 50 fold), while there was no effect on the *G*. *vaginalis* load in 8 (42%) patients. In one patient there was a reduction day 3 and 5 followed by an increase in load. There was no difference in the treatment effect related to the single-dose therapy versus the 7-day regimen.

In the 11 patients with *G*. *vaginalis* eradication or significant reduction, *A*. *vaginae* was also present in 10 and eradicated in all. Of the eight patients without treatment effect on *G*. *vaginalis*, six were also *A*. *vaginae* positive and three remained unchanged during treatment while in three, *A*. *vaginae* was eradicated (in two only temporary). Of the eight patients without treatment effect on *G*. *vaginalis*, seven were positive and effectively treated for *M*. *genitalium* or *C*. *trachomatis*.

### Specific qPCR compared to 16S sequencing

#### *M*. *genitalium* and *C*. *trachomatis*

By qPCR, 57 samples were *M*. *genitalium* positive. However, only 10 (18%) of these were detected in the microbiota analysis. By qPCR, the median organism load was 394 for the sequence-positive and 23 for the sequence–negative (p<0.00001, Mann-Whitney).

For *C*. *trachomatis*, 36 samples were qPCR positive and eight (22%) samples from five patients were sequence positive. The median organism load for the eight sequence-positive samples was 168000 while it was 854 for the 28 sequence negative samples. All of the sequence-positive samples were from day 0, 1 or 3.

#### *Ureaplasma* spp

In total, 53 patient samples were *U*. *urealyticum* positive by qPCR, 3/53 were also *U*. *parvum* positive. Of the 53 *U*. *urealytium* qPCR positive, 29 (55%) were sequence positive. The median load (qPCR) was 274 for the sequence positive and 7.5 for the sequence negative (p<0.00001, Mann-Whitney). A total of 188 samples were *U*. *parvum* positive by qPCR and of these, 156 (83%) were sequence positive. The median qPCR load was 59 for the sequence positive and 17.5 for the sequence negative (p<0.002, Mann-Whitney).

#### Specific qPCR and antimicrobial treatment

**Fig 6:**
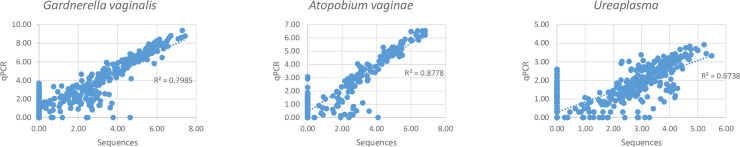
Correlation between sequences and qPCR for *G*. *vaginalis*, *A*. *vaginae* and *Ureaplasma*. The bacterial load determined by rarefied sequence number multiplied by total bacterial load (sequences) versus the load determined by specific qPCR for *Gardnerella vaginalis*, *Atopobium vaginae* and *Ureaplasma*. Log scale. *Ureaplasma* is *U*. *parvum* and *U*. *urealyticum* combined.

The preparation of samples for sequencing at the MiSeq platform includes a number of normalization steps that, potentially, could skew the distribution of species in the sequenced samples. To address this, we compared the bacterial load as determined by species-specific qPCR with the total bacterial load by qPCR multiplied by the relative abundance of sequences for the individual species. The relation between qPCR and normalized sequences for *G*. *vaginalis*, *A*. *vaginae* and *Ureaplasma* is shown in Fig 6 (log 10 data).

For the more abundant species, *G*. *vaginalis* and *A*. *vaginae*, there was a relatively good correlation between the load determined by specific qPCR and the load determined by sequencing. For *Ureaplasma*, where the load was approximately 100 fold lower, the correlation was relatively poor, in particular for samples with high bacterial load and low load of *Ureaplasma*. For all three species, a considerable number of samples showed a qPCR of 100–1000 copies, but zero sequences, probably reflecting that specific PCR is more sensitive than a broad-range 16S PCR.

#### Ureaplasma

Twelve patients (23%) were qPCR positive for *U*. *urealyticum* at day zero or one, and all twelve were treated with azithromycin. For eight of these patients (67%), *U*. *urealyticum* was eradicated, while no effect was observed in three patients. In one patient the load was too low for evaluation. *U*. *parvum* was only eradicated in one of the four patients who were also *U*. *parvum* positive.

Twenty-eight patients (54%) were *U*. *parvum* positive at day at 0 or 1 and were treated with azithromycin. In one of these, the load was too low for evaluation. In 25 of the 27 patients (93%), *U*. *parvum* persisted as determined by qPCR and only in two patients (7%) (#9 and #27, both CST-IV) *U*. *parvum* was eradicated by azithromycin.

The six patients treated with tetracyclines were all *U*. *parvum* positive at day 0 or 1. Five of these (83%) demonstrated eradication, while one had a temporary reduction in the organism load.

For 274 of the patient samples, both qPCR results and a Nugent score were obtained. The load of *U*. *parvum*, as determined by qPCR, was significantly related to the Nugent score (p<0.0045, Pearson), whereas there was no statistically significant relation between Nugent score and load of *U*. *urealyticum*. (p = 0.092).

The 25 patients where *U*. *parvum* was persistent were equally distributed regarding CSTs (CST-I: 6/14 (43%), CST-III: 12/22 (55%), CST-IV: 7/16 (44%)) or concomitant use of metronidazole (10 treated with metronidazole, 15 without treatment).

#### *G*. *vaginalis*, *A*. *vaginae* and *Prevotella*

Using the concept of conversion from relative abundance to absolute counts, there was no significant difference in the load of *G*. *vaginalis* or *A*. *vaginae* for metronidazole treated patients at any time-point. In contrast, the genus *Prevotella* (combined data for all *Prevotella* species) showed a considerable reduction following administration of metronidazole, but only significant for day 5 and day 8, both p<0.01(Wilcoxon).

#### *M*. *genitalium*, *C*. *trachomatis* and BV

At day 0, 32 patients were *M*. *genitalium* positive, 10 were *C*. *trachomatis* positive while two patients were positive for both of these species. Eight patients were negative for both.

Of the 32 *M*. *genitalium* positive patients, 16 (50%) were BV positive in the clinic by Amsel, while five (50%) of the 10 *C*. *trachomatis* positive had BV. None of the two double infected patients was treated for BV (one negative, one without gynecological examination). Of the eight patients without Ct and Mg, three (38%) had BV.

Neither *C*. *trachomatis* nor *M*. *genitalium* were related to any specific community state type. In CST-I, CST-III and CST-IV 21%, 13% and 31%, respectively were *C*. *trachomatis* positive, for *M*. *genitalium* the corresponding figures were 57%, 50% and 75%.

## Discussion

The vaginal microbiota, particularly in women with BV, is relatively complex and with considerable differences between individuals. We found 615 different taxa in vaginal swabs from 62 women. Despite this high number of different species, only 33 taxa represented more than 1 ‰ of the sequences and combined covered almost 98% of the sequences. In any environment, administration of antibiotics may not only eradicate the intended species, but may also affect other bacteria with unknown roles in the microbiota. Thus, we aimed to longitudinally evaluate the effect of antimicrobial treatment on the vaginal microbiota to study its resilience. We were particularly interested in the different spectrum of azithromycin and tetracyclines to inform the current discussions recommending to abandon azithromycin treatment for syndromic management of non-gonococcal urethritis and cervicitis in favor of doxycycline [30,31].

It is well known that risk of acquisition of several STIs is increased in the presence of BV [12,13] but acquisition of a bacterial STI may also lead to disturbed vaginal microbiota [12]. This was reflected in the high proportion of women in the present study having BV by Amsel criteria at inclusion and made comparison of a “clean” macrolide versus tetracycline treatment difficult, as it was considered unethical to withhold BV treatment.

Despite this limitation, we saw a surprising resilience of the microbiota regardless of the treatment provided. Even though treatment efficiently eradicated *C*. *trachomatis* and *M*. *genitalium*, we were not even able to document a statistically significant decrease in the total bacterial load as measured by qPCR. In women with BV, a temporary decrease in diversity was observed in connection with the metronidazole treatment, but at the end of the 26 day observation period, most women had their pre-treatment level of diversity.

The vaginal microbiota in both controls and patients was in accordance with earlier described community state types (CST-I–CST-V) except that we did not find patients with a microbiota dominated by *L*. *gasseri* (CST-II) or by *L*. *jensenii* (CST-V), although a few samples had high abundance of *L*. *jensenii* and a single of *L*. *gasseri*.

This is somewhat in contrast to earlier findings; Ravel *et al* found CST-II in 6% and CST-V in 5% of patients from the US, and even higher fractions in patients who identified themselves as white[2], while Virtanen *et al* found both *L*. *gasseri* and *L*. *jensenii* to be dominant in each 4% of the samples from Nordic women [6].

However, the relatively low number of women in the present study could also explain the inability to identify CST-II and CST-V.

In the controls, the 10 most abundant species were rather stable over time in nine out of ten women and this was the case not only in *L*. *crispatus* dominated CST-, but also in CST-III dominated by *L*. *iners*.

In the patients, treatment with azithromycin was very effective in eradicating the intended targets *M*. *genitalium* and *C*. *trachomatis*, but had little impact on the vaginal microbiota and did only change the CSTs in a few patients. In contrast to azithromycin, metronidazole had a significant effect on the diversity of the vaginal microbiota (Fig 4). Still, in approximately 50% of the patients, metronidazole did not change the CST-IV status. In another 10 patients the microbiota rapidly changed to CST-III. Interestingly, two patients without metronidazole treatment also changed from BV to CST-III. Such an increase in *L*. *iners* following treatment with metronidazole has also been seen in other studies [32,33]. Jacobsson and Forsum suggested that *L*. *iners* dominance could be a transitional stage between abnormal and normal microbiota [34]. This was, however, not supported in the present study, at least during the 26 days observation period, as not a single treated BV patient changed from CST-IV over CST-III to CST-I.

Metronidazole is one of the most commonly used antimicrobial treatments for BV, however, the cure rate is rather low [35] and recurrence is frequent [36]. *G*. *vaginalis* is one of the prominent vaginal bacteria associated with BV. We found that metronidazole only eradicated or significantly reduced *G*. *vaginalis* in 58% of the treated patients, and in these patients, other BV associated bacteria such as *A*. *vaginae* were also eradicated. In those patients where *G*. *vaginalis* was not affected, other anaerobic bacteria were eradicated in half the patients, demonstrating that the microbiota was affected to some extent by the metronidazole treatment, even if *G*. *vaginalis* did not respond. In the remaining half of the patients, none of these anaerobic bacteria was eradicated. This could be due to biofilm formation protecting *G*. *vaginalis* as well as other members of the biofilm, but the persistence of *G*. *vaginalis* could also reflect true metronidazole resistance which has been reported in >50% of endometrial *G*. *vaginalis* and *A*. *vaginae* isolates [37].

The persistence of *G*. *vaginalis* could also be caused by patients not using the prescribed metronidazole. However, all patients carefully collected and submitted samples. Further, in most of the patients without effect on *G*. *vaginalis*, *M*. *genitalium* or *C*. *trachomatis* were eradicated, demonstrating that the antibiotics prescribed for STIs were used. We therefore find it likely that most or all patients did comply with the prescribed treatment.

In contrast to the present study, Mayer *et al* [38] found a rapid and profound shift in the vaginal microbiota after treatment for BV by specific qPCRs. In particular *Leptotrichia/Sneathia* and *Megasphaera* were rapidly cleared but significant reduction in *G*. *vaginalis* was also observed in all patients. However, the majority of the women received local metronidazole which may have produced higher concentrations locally explaining the difference. On the other hand oral and local metronidazole is considered equally clinically efficient [39]. Still, the median load of *Gardnerella* was not significantly reduced from day 0 before treatment to any of the following samplings when all patients receiving metronidazole were compared. In the patients where *G*. *vaginalis* was unaffected by metronidazole, the load actually increased from day zero, probably reflecting that competitor species were reduced or eradicated. The increasing load in these samples could mask a decreasing trend in patients with metronidazole susceptible *G*. *vaginalis*.

In both controls and patients we saw changes in the microbiota and increasing diversity related to bleeding. In most cases, the microbiota rapidly returned to the CST observed before the bleeding incident. In particular, the taxon *Enterobacteriaceae* was related to periods of vaginal bleeding both in patients and controls. This is consistent with an earlier study that found *E*. *coli* associated with the menstrual phase [40]. Bleeding *per se* and the following shift in pH and enrichment of iron and other nutrients could promote such flare up of *Enterobacteriaceae*. Another explanation could be a depletion of lactobacilli. A bactericidal activity of genital tract secretions against *E*. *coli* has earlier been described and Kalyoussef *et al* found proteins secreted from different lactobacilli to be associated with this bactericidal activity [41]. A later study found that supernatant of *L*. *crispatus* cultures could inhibit growth of *E*. *coli*, though some effect of supernatant of *L*. *iners* and *G*. *vaginalis* also was observed [42]. Other studies have found other bacteria related to menses, Srinivasan *et al* observed an increase of *G*. *vaginalis* [11]. Interestingly, Gajer *et al* [10] found no significant difference in Shannon diversity index in relation to menstrual period when analyzing 32 women twice weekly for 16 weeks but found the highest rate of change in the diversity during menses.

Treatment with metronidazole reduced, temporarily, the diversity (Fig 4) as the abundance of *L*. *iners* increased while *Prevotella* decreased. This is consistent with earlier findings by Verwijs *et al* [35] who found a reduction in BV associated taxa combined with an increase of lactobacilli. However, in contrast to the present study, they found almost 1 log reduction in the total bacterial load following treatment with metronidazole.

While we found tetracyclines effective against both *U*. *parvum and U*. *urealyticum*, the response to azithromycin differed between the two species. *U*. *urealyticum* was eradicated in 67% of the treated patients, whereas *U*. *parvum* was eradicated in only 7% of the treated patients. Interestingly, the load of *U*. *parvum* was positively correlated to the Nugent score, i.e. related to CST-IV. This is in accordance with our previous findings in men where the load of BV associated bacteria was positively correlated with the ureaplasma load [43]

The acidic environment created by the vaginal lactobacilli, particularly *L*. *crispatus* [44] could diminish the activity of azithromycin. However, we did not see more ureaplasma persistence in the CST-I dominated patients, on the contrary, patients with CST-III and CST-IV showed a higher proportion of persistent *U*. *parvum* than CST-I dominated patients.

Few studies have used standardized methods for *in vitro* MIC determination of azithromycin and distinguished between the two species. Valentine-King and Brown found *U*. *parvum* to have low resistance to azithromycin (MIC of ≤0.5–2 μg/ml) for 60 isolates from the United States and only slightly higher MICs for *U*. *urealyticum* (MIC of ≤0.5–4 μg/ml) [45]. A similar trend towards higher MIC for *U*. *urealyticum* was reported from China [46], and a study analyzing ureaplasmas from neonates from the UK found no resistance to macrolides and no different activity of azithromycin, but a significantly higher erythromycin MIC for *U*. *urealyticum* [47]. However, these *in vitro* studies may not reflect the *in vivo* activity, where factors like biofilm or pH may influence the activity of antibiotics. To our knowledge, the differential in vivo response to azithromycin has not previously been investigated.

### Strengths and limitations

Strengths of the present study includes repeated sampling of patients and controls over a 26 day period and sequencing data supplemented with qPCR to allow a better quantitation. A limitation is the size of the study population where the 491 sequenced samples represent only 52 patients and 10 controls. With this limited number of participants more subtle changes in the microbiota may have been missed. More participants would possibly have allowed stronger quantitative conclusions.

In conclusion, the more dominant part of the vaginal microbiota was found to be remarkably resilient after treatment with azithromycin and tetracyclines as treatment only changed the CST in a few patients. Only women receiving metronidazole changed from CST-IV to CST-III, but only in 50% of the treated patients, and the effect on key species as *G*. *vaginalis* and *A*. *vaginae* was limited and temporary. A differential activity of azithromycin against *Ureaplasma* spp. was observed which should be confirmed in future studies

## Supporting information

S1 Table Bacterial species identified as contaminants. (DOCX)

S2 Table Spreadsheet showing the distribution of rarefied sequences in patients and controls. Column A: Sample name. Column B: Patient code. Patients treated with antibiotics: 8–200. Controls: 207–226. Column C: Sampling number. Column D: Sampling day. Column AL: Sum of all species constituting < 1‰ of the sequences. Column E–AK and AM—WV: Bacterial species in descending order.
(XLSX)

S1 Fig Bacterial load (log10) in patients for day 0 to day 26. (DOCX)
