# Supplementary material for: Changes in the vaginal microbiota following antibiotic treatment for Mycoplasma genitalium, Chlamydia trachomatis and bacterial vaginosis
Source: PLoS One. 2020 Jul 28;15(7):e0236036. doi: 10.1371/journal.pone.0236036 (PMC7386580; doi:10.1371/journal.pone.0236036)
Supplement: S1 Table — (DOCX) [file pone.0236036.s001.docx]

S1 Contaminating bacterial species

*Acinetobacter baumannii*

*Acinetobacter calcoaceticus*

*Acinetobacter haemolyticus*

*Bacillus cereus*

*Burkholderia caledonica*

*Caldimonas hydrothermale*

*Cloacibacterium normanense*

*Comamonas denitrificans*

*Cupriavidus basilensis*

*Diaphorobacter nitroreducens*

*Dyella terrae*

*Propionibacterium acnes*

*Pseudomonas fluorescens*

*Ralstonia solanacearum*

*Rothia mucilaginosa*

*Sediminibacterium salmoneum*

*Sphingomonas oligophenolica*

*Streptococcus oralis*
