# Supplementary material for: Changes in the vaginal microbiota following antibiotic treatment for Mycoplasma genitalium, Chlamydia trachomatis and bacterial vaginosis
Source: PLoS One. 2020 Jul 28;15(7):e0236036. doi: 10.1371/journal.pone.0236036 (PMC7386580; doi:10.1371/journal.pone.0236036)
Supplement: Supplementary file 2 [file pone.0236036.s003.docx]

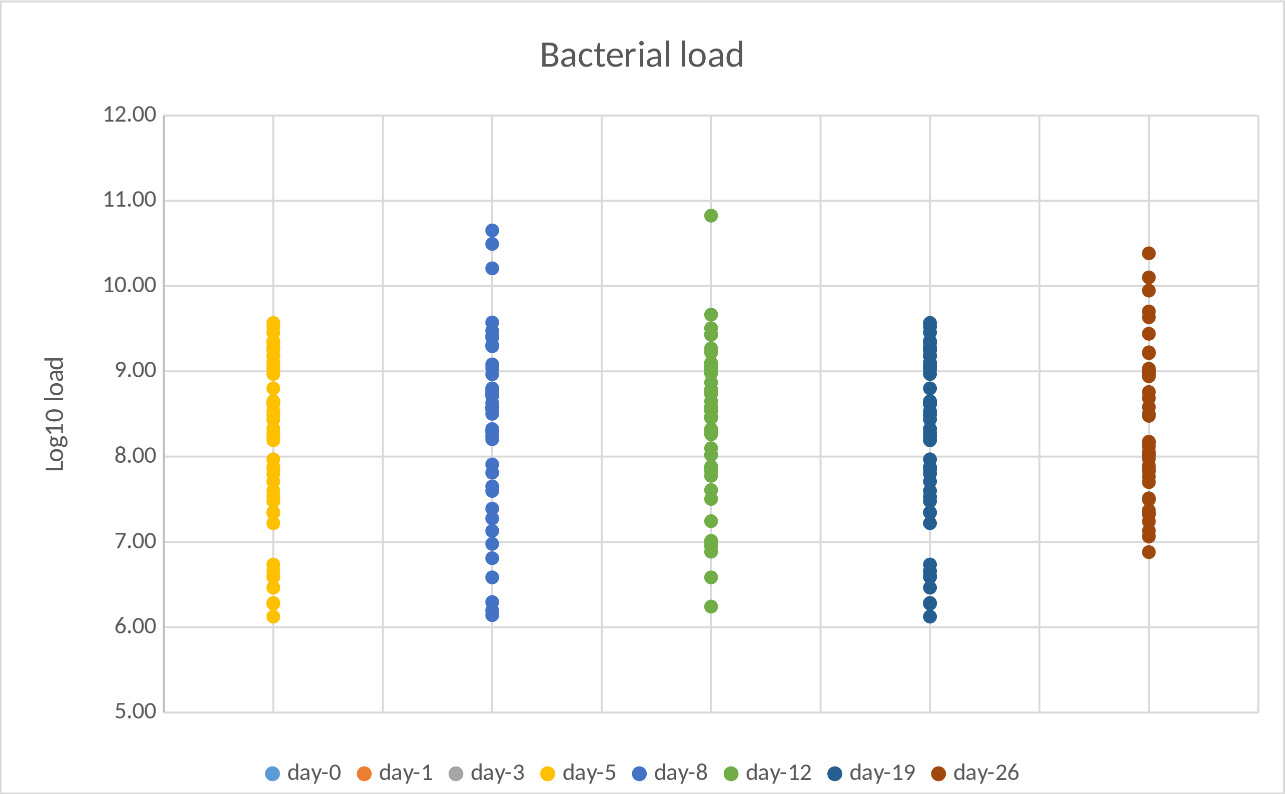
Bacterial load
Log10 load
12.00
11.00
10.00
9.00
8.00
7.00
6.00
5.00
day-0
day-1
day-3
day-5
day-8
day-12
day-19
day-26
